# Design and Mechanical Evaluation of a Capacitive Sensor-Based Indexed Platform for Verification of Portable Coordinate Measuring Instruments

**DOI:** 10.3390/s140100606

**Published:** 2014-01-02

**Authors:** Agustín Brau Avila, Jorge Santolaria Mazo, Juan José Aguilar Martín

**Affiliations:** Department of Design and Manufacturing Engineering, University of Zaragoza, María de Luna 3, Zaragoza 50018, Spain; E-Mails: jsmazo@unizar.es (J.S.M.); jaguilar@unizar.es (J.J.A.M.)

**Keywords:** coordinate metrology systems, portable coordinate measuring machine, indexed metrology platform

## Abstract

During the last years, the use of Portable Coordinate Measuring Machines (PCMMs) in industry has increased considerably, mostly due to their flexibility for accomplishing in-line measuring tasks as well as their reduced costs and operational advantages as compared to traditional coordinate measuring machines (CMMs). However, their operation has a significant drawback derived from the techniques applied in the verification and optimization procedures of their kinematic parameters. These techniques are based on the capture of data with the measuring instrument from a calibrated gauge object, fixed successively in various positions so that most of the instrument measuring volume is covered, which results in time-consuming, tedious and expensive verification procedures. In this work the mechanical design of an indexed metrology platform (IMP) is presented. The aim of the IMP is to increase the final accuracy and to radically simplify the calibration, identification and verification of geometrical parameter procedures of PCMMs. The IMP allows us to fix the calibrated gauge object and move the measuring instrument in such a way that it is possible to cover most of the instrument working volume, reducing the time and operator fatigue to carry out these types of procedures.

## 1. Introduction

An important group of coordinate metrology systems is that consisting of portable measuring instruments such as articulated arms coordinate measuring machines (AACMM) and laser trackers (LT). The use in industry of these types of portable equipment has increased considerably during the last years, mostly due to their flexibility for accomplishing in-line measuring tasks as well as their reduced costs and operational advantages as compared to traditional coordinate measuring machines. However, their operation has a significant drawback derived from the techniques applied in the verification and optimization procedures of their kinematic parameters. So far, the only standards developed for AACMM are the ASME B89.4.22-2004, the draft ISO/CD 10360-AA and the VDI guideline 2617-9, and for LT, the ASME B89.4.19 and the VDI guideline 2617-10 [1–5]. Currently, these techniques are based on the capture of data with the measuring instrument from a calibrated gauge object, such as a ball bar gauge, successively fixed in various positions so that most of the instrument measuring volume is covered [6]. In each position, a support is used to rigidly fix the gauge object at different heights and orientations with respect to the measuring instrument. These changes of position result in a time-consuming, tedious and expensive verification procedure. Thus, the design of novel devices that allow the simplification of the mentioned techniques is of paramount concern for research in coordinate metrology [7].

In recent years many authors have developed new novel devices to carry out the verification and optimization procedures of PCMMs, particularly of AACMM and LT. In [8] a new method to estimate the uncertainty of a measuring arm using a tridimensional gauge is presented. This method consists of a flat plate with nine spheres fixed at three different heights with respect to the metallic surface of the plate. Then the spheres' centers are measured with the measuring arm at different locations and orientations, and the distances between the sphere centers are compared to the nominal between the spheres' centers is calculated and compared to the nominal distances measured with a Coordinate Measuring Machine (CMM). The same author developed a virtual spheres plate gauge to check the performance of AACMMs consisting of 16 groups of four conic holes placed on an aluminum plate to determine 16 virtual spheres. The gauge was placed at three positions within the AACMM work volume to take coordinates' points at each hole with a spherical rigid probe and as in [9], these points were also fitted to spheres and the distances between the spheres' centers were calculated and compared to the nominal distances measured with a CMM [10]. In [11] the influence of the contact force by the operator on the performance of AACMMs was measured by means of a contact force sensor developed by the authors in which the contact force was proved to be a main factor influencing the AACMMs performance. In [12] the optimal measurement area and a spatial error distribution model of an AACMM is determined by using a Support Vector Machine approach. Kovac and Frank [13,14] proposed the use of a high precision gauge instrument for the verification and calibration of AACMMs. Their work focused on the development, manufacturing and characterization of this instrument to use it in parameter identification procedures and evaluation tests of AACMMs in laboratories, determining the gauge uncertainty due to its main influence variables. Santolaria proposed a calibration process based on the Denavit-Hartenberg kinematic model parameters. These parameters are optimized measuring a calibrated ball gauge bar positioned at different orientations and positions of the AACMM work volume [15–17]. Ouyang proposed a laser tracker calibration method using coordinate measuring machines. Using this method, a commercial laser tracker was calibrated and angular errors were found to be the key error source [18]. In [19,20] the authors present the development of a metrological model to identify the kinematical parameters of a measuring arm as well as the errors associated with its measurements. Moreover, using the metrological model information, the authors developed a virtual kinematic model with CATIA software, which objective was to assess the measuring performance of the measuring arm without physically using the AACMM. González *et al*. [21] presented a virtual circle gauge method to evaluate for the assessment of AACMM throughout its measuring volume.

In most of the works found in the literature, a common task during the verification or calibration of PCMMs depends on the necessity of locating at different heights and orientations a calibrated gauge object throughout the working volume of the measuring instrument. As mentioned before, this result in a time-consuming, tedious and expensive verification and calibration procedures, that increases the costs of carrying out these types of procedures which industries are not always willing to assume.

In this work, an explanation of a novel design of an indexed metrology platform is presented and the most important aspects of its sensors, mechanisms and mechanical components are explained. The use of six capacitive sensors in the IMP and the paramount importance of this measurement device during the verification procedures of PCMMs is explained. Finally, the mechanical repeatability achieved by the IMP with the kinematic coupling arrangement of spheres and cylinders is highlighted.

In [22] a brief explanation of the main components of the mechanisms composing the IMP is presented. Nevertheless, the results of the evaluation of the mechanical positioning repeatability obtained by means of kinematic couplings is not discussed as well as the assessment and importance of the capacitive sensors during the verification procedures of PCMMs. Finally, as mentioned, a complete explanation of the function of the IMP mechanisms and all its components is presented in this paper.

The main purpose of the IMP is to drastically simplify the verification and identification procedures of PCMMs through an alternative to the typical procedures by inverting the roles, that is, fix the calibrated gauge object and move the measuring instrument in such a way that it is possible to cover most of the instrument working volume. To this end, the PCMM is fixed to the IMP, so it can vary its relative position with respect to the calibrated gauge object with a high mechanical position repeatability knowing its position and orientation in the global reference system of the lower platform. This way, the time and effort needed to carry out this type of procedure for PCMMs (*i.e.*, AACMM) can be reduced from approximately two days to only three hours by eliminating the necessity of moving support of the calibrated gauge object around the measuring instrument.

## 2. Mechanical Components of the Indexed Metrology Platform

**Figure 1.**
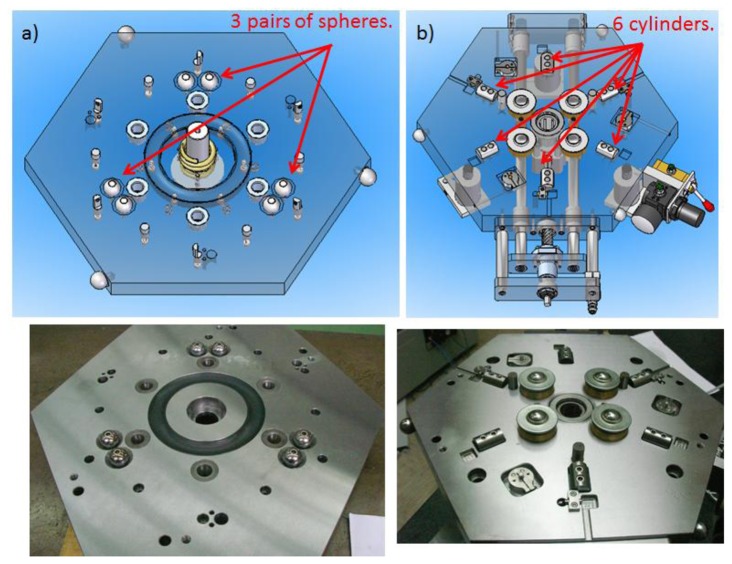
(**a**) Pairs of spheres in upper platform; (**b**) Cylinders located at fixed lower platform.

The IMP consists of a mobile hexagonal upper platform and a hexagonal fixed lower platform of dimensions 398.5 mm × 345 mm, designed in such way that the upper platform rotates around the fixed lower platform and descends every 60°, thus having six possible different positions of the upper platform with respect to the lower platform. Moreover, to cancel the degrees of freedom (DoF) and to ensure a good mechanical repeatability of the upper platform with respect to the lower platform, an arrangement of spheres and cylinders kinematic couplings is utilized. In the upper and lower platform three pairs of spheres are located at 120° between them and six cylinders are located at 60° between them respectively as shown in Figure 1.

**Figure 2.**
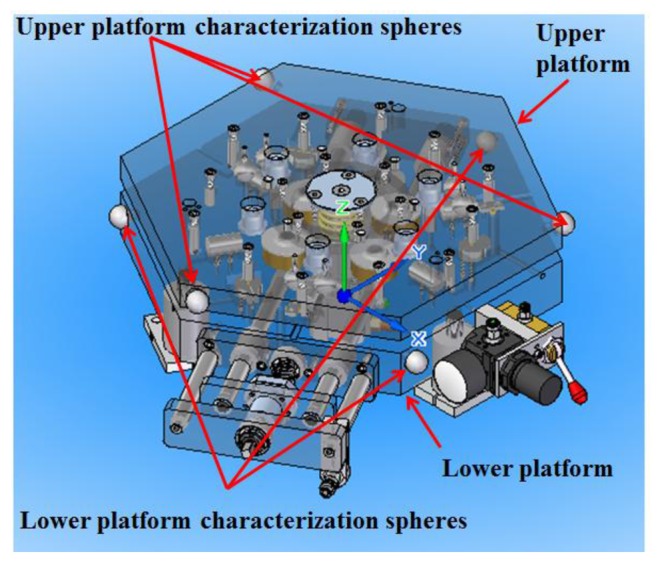
Upper and lower platform characterization spheres.

To determine the upper platform and lower platform reference systems, three characterization spheres are located on the sides of the platforms and measured with a CMM (Figure 2). These spheres are of great importance because they will allow us to express coordinates of measured data in the fixed lower platform global coordinate system during the verification of PCMMs.

**Figure 3.**
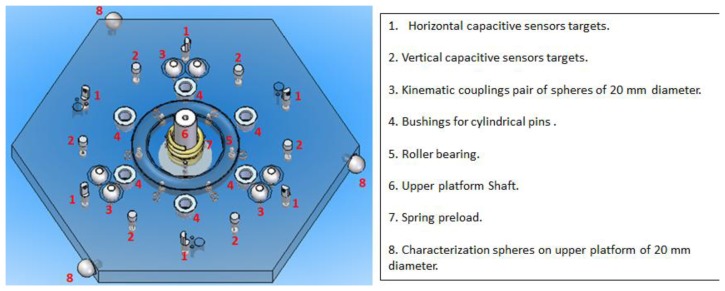
Mobile upper platform mechanical components.

**Figure 4.**
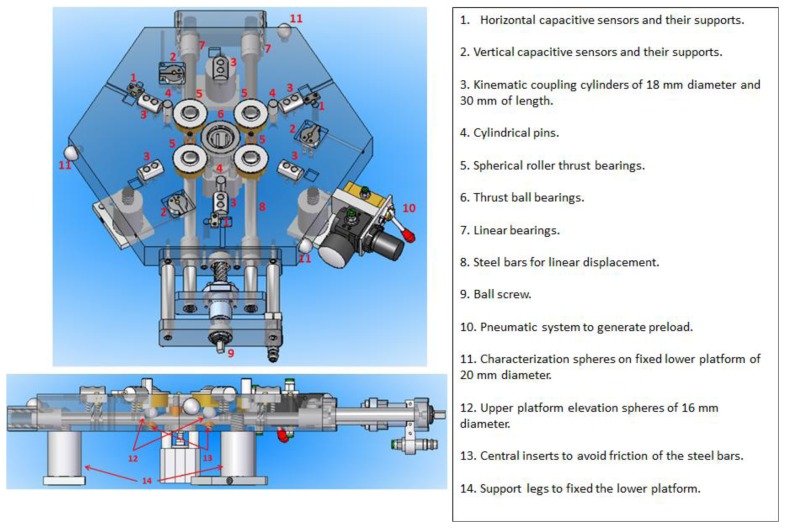
Fixed lower platform mechanical components.

The upper and lower platforms consist of the following main mechanical components shown numerically in Figures 3 and 4 respectively.

Kinematic couplings have widely been used for positioning one rigid body with respect to another with a high repeatability in applications such as metrology, manufacturing, fixturing and material handling. Nevertheless, as far as we know, they have not been used in PCMMs metrology applications, in which their high repeatability and interchangeability are of great importance [23,24].

**Figure 5.**
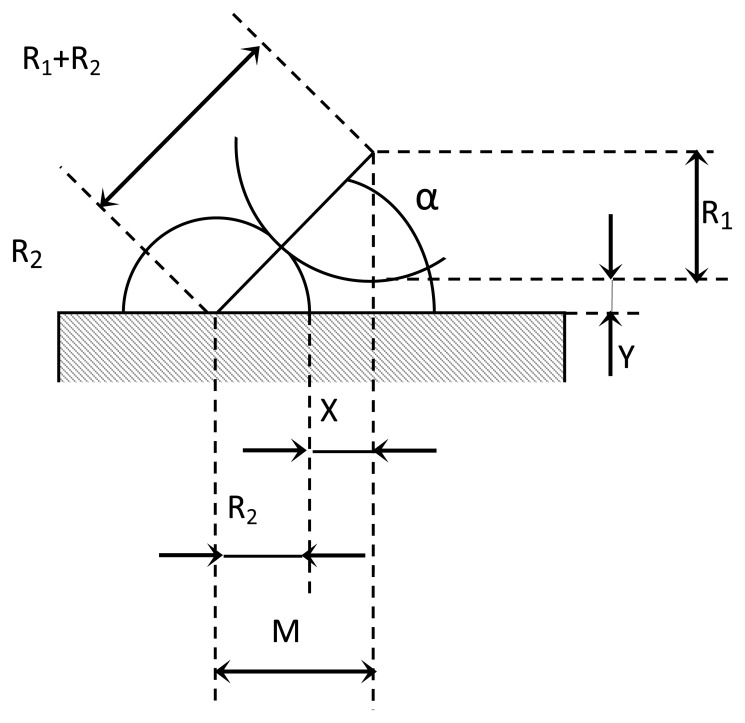
Kinematic coupling variables design.

In order to use the IMP in the verification procedure of PCMMs, a high mechanical positioning repeatability of the upper platform with respect to the lower platform has to be achieved. To this end, the arrangement of the kinematic couplings used in this work was selected based on a previous work where a high positioning mechanical repeatability was obtained [25]. In that arrangement the kinematic couplings spheres and cylinders were inserted half their height into the upper and lower platforms respectively. In Figure 5 a frontal view of a sphere and cylinder of the kinematic coupling is shown; where variables *R_1_* and *R_2_* correspond to the sphere and cylinder radius respectively, *α* is the angle formed from the lower platform surface to the straight line connecting the sphere and cylinder centers, *M* represents the vertical distance of the right triangle formed between the spheres and cylinder centers and *X* is the difference between parameter *M* and radius *R_2_*.

The following equalities can be established from Figure 5 (Equations (1) and (2)):

(1) $$X=M-R_{2}$$

(2) $$M=\sqrt{{\left(R_{1}+R_{2}\right)}^{2}-{\left(R_{1}+Y\right)}^{2}}$$

By substituting Equation (1) in Equation (2) we obtain Equation (3):

(3) $$X=\sqrt{R_{2}^{2}2R_{1}R_{2}-Y^{2}-2R_{1}Y}-R_{2}$$

Once the value of *X* has been calculated, it is possible to obtain *α* by means of the following (Equation (4)):

(4) $$\alpha =\arcsin\left(\frac{R_{1}+Y}{R_{1}+R_{2}}\right)$$

In [26–30] it is established that for balanced stiffness in all directions in the kinematic coupling, the contact force vectors should intersect the plane of coupling action at an angle of 45°. In our case, this angle is represented by the parameter α. To find the searched variables that satisfy this condition, initial values were assigned to the sphere and cylinder radius, *R_1_* and *R_2_*, and to *Y,* obtaining the following values: *R_1_* = 10 mm, *R_2_* = 9 mm, *Y* = 3.43 mm, *M* = 13.44 mm, *X* = 4.44 mm and *α* = 44.97°.

### 2.1. Elevation Mechanism

**Figure 6.**
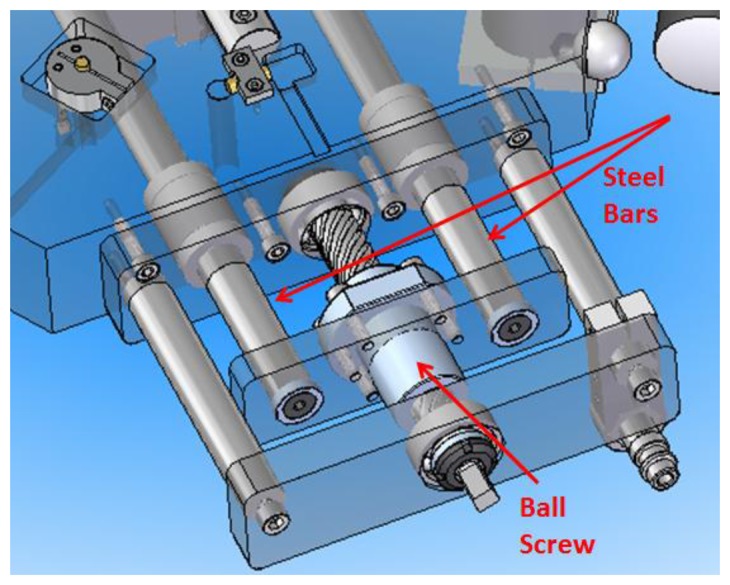
Ball screw and steel bars of the elevation mechanism.

**Figure 7.**
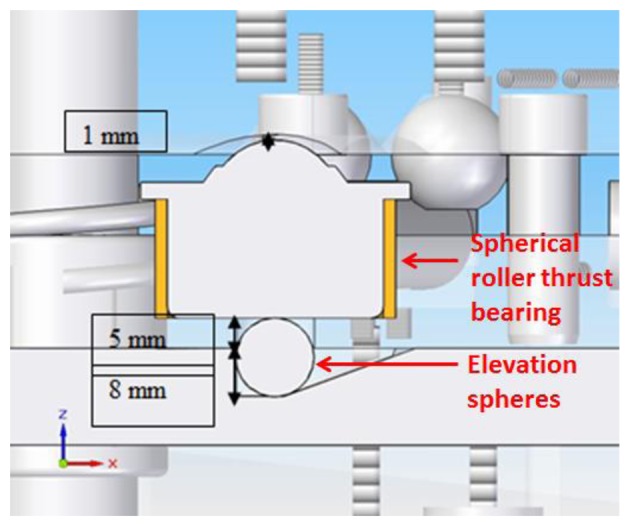
Detail view of steel bar, elevation spheres and spherical roller thrust bearing.

As explained before, with the arrangement of the kinematic coupling selected it is possible to position the upper platform with respect to the lower platform in six different positions with the kinematic coupling being the only support elements between platforms. This six positions are manually interchangeable by the operator and the change of one position to another is achieved by means of an elevation mechanical system composed of a ball screw, two steel bars with linear displacement; within the steel bars two 13 mm spheres are housed beneath four spherical roller thrust bearings protruding from a cylindrical hole in the center of the spherical roller thrust bearings seats, as shown in Figures 6 and 7.

**Figure 8.**
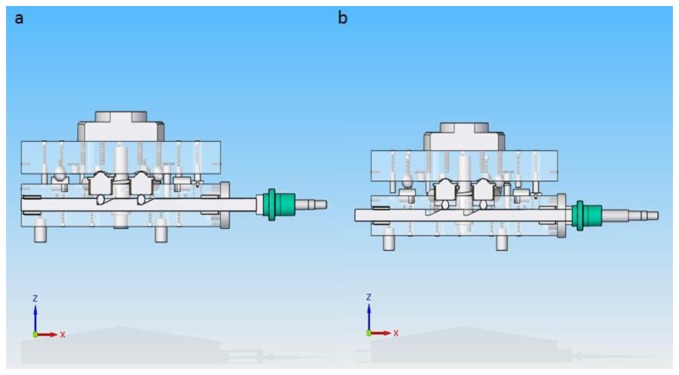
(**a**) Upper platform before elevation (close position); (**b**) Upper platform after elevation (open position), ready to change to a new position.

The elevation of the upper platform is realized by turning the ball screw clockwise 180°, generating a linear displacement of the two steel bars that elevate the housed elevation spheres which at the same time push up the spherical bearings and then the upper platform. In Figure 8a,b the platform before and after elevation respectively is shown.

**Figure 9.**
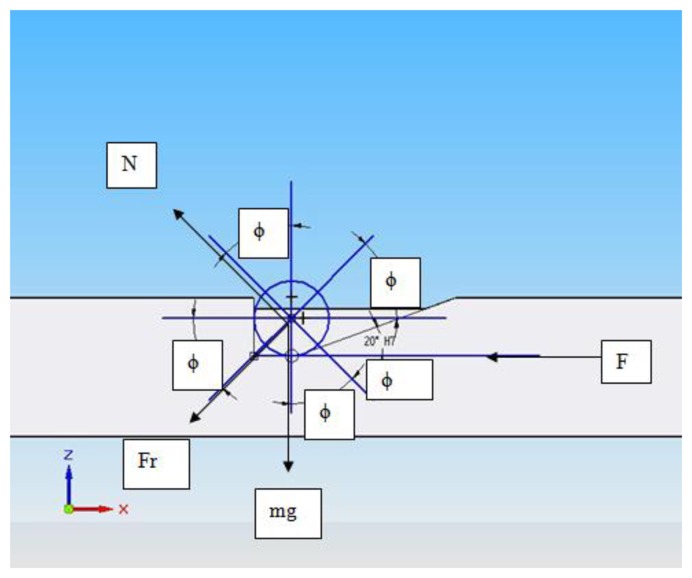
Scheme of equilibrium of forces to determine the horizontal force needed to elevate one sphere.

Moreover, the steel bars feature a ramp that allows the elevation spheres to slide along them. To determine the angle of the ramp as a function of the slope inclination, the horizontal force required to elevate a sphere was calculated. In Figure 9 the equilibrium of forces to determine the horizontal force is represented.

From Figure 9 the following equations can be determined (Equations (5)–(7)):

(5) Fr=μ⋅N

(6) Fcosϕ−Fr⋅mg⋅senϕ=0

(7) N−mg⋅cosϕ−Fsenϕ=0

then by solving *F* we can obtain the force to elevate the upper platform as follows (Equation (8)):

(8) $$F=\frac{mg(\mu \cdot \cos\phi +\text{sen}\phi )}{\left(\cos\phi -\mu \cdot \text{sen}\phi \right)}$$

where *m* is the total mass to be elevated, equals to the sum of the weight of the upper platform (10 kg) and an AACMM or LT (11 kg. approximately); *μ* (0.57) represents the friction coefficient between the steel bars and the elevation spheres; *g* (9.81 N/Kg) represents the gravity of Earth constant, *N* the normal force and *Fr* represents the frictional force between the elevation spheres and the ramp; it must be noted that when the elevation spheres slide through the ramp, there is no contact between the spheres and the vertical wall, so there is no frictional force between them. The force obtained in Equation (8) constitutes the force needed to elevate the upper platform and the measuring instrument to be verified. Nevertheless, since there are four elevation spheres, the total force calculated in Equation (8) must be divided by four in order to know the force in each elevation sphere. In Table 1 the different values of *ϕ* with the corresponding force *F* needed to elevate the platform and linear displacement of the steel bars are shown.

From the values of Table 1, it can be observed that as the inclination angle of the slope increases (*ϕ*), the force needed to elevate the weight also increases and the linear displacement of the steel bars decreases. The minimum linear displacement required of the steel bars in order to elevate the spheres through the ramp as shown in Figure 8b is 17 mm. Moreover, it must be noted that as the angle increases the force needed to elevate the weight drastically increases and the displacement of the steel bars is shorter than the minimum required. For this reason an inclination angle of the slope of 20°, with a force of 48.095 N and a displacement of 17.56 mm was selected. Once the inclination angle was selected, a ball screw type that would allow at least the minimum linear displacement with a 180° clockwise turn was required. A ball screw of 16 mm diameter with six inputs or threads and a lead pitch of 50 mm was selected. This means that for every 180° turn the linear displacement would be 25 mm, fulfilling one of the most important requirements for the selection of the ball screw.

### 2.2. Rotation and Descend Mechanisms

**Figure 10.**
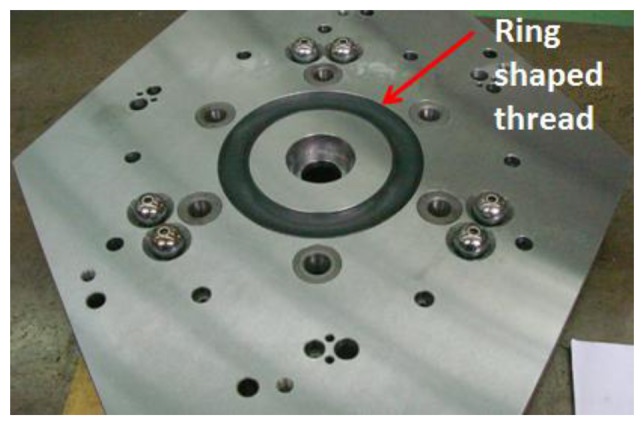
Ring shaped ring to ensure a rotational movement.

Once the upper platform has been elevated, it can rotate 60° around its central shaft to a new position. This is achieved by means of the spherical roller thrust bearings that are guided by a ring shaped thread (Figure 10) which ensures a rotational movement.

**Figure 11.**
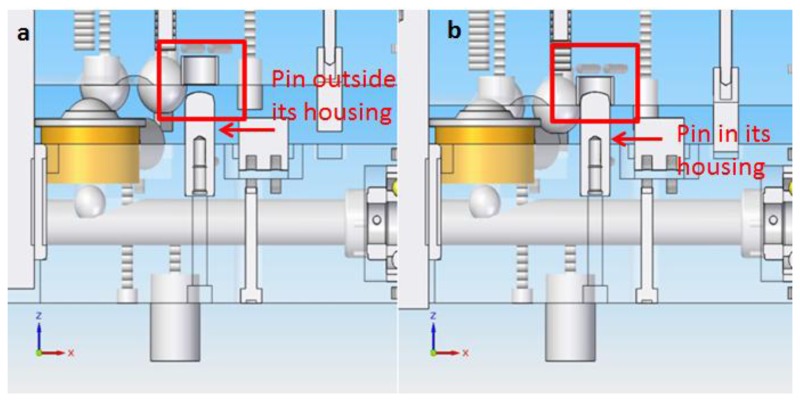
(**a**) View of pin outside its housing; (**b**) View of pin inside its housing.

When the upper platform has been manually rotated 60° to a new position, the ball screw is turned back 180°, which will cause the steel bars, elevation spheres and spherical roller thrust bearings to return to their starting position and the upper platform to descend as shown in Figure 8. As mentioned before, since the upper platform rotates 60° to a new position, thus there are six possible different positions of the upper platform with respect to the lower platform, in which the contact points of the kinematic couplings between the cylinders and spheres are the only physical contact in order to cancel the DoF of the IMP. To make sure that the upper platform exactly rotates 60°, three pins are fastened with their corresponding housings to the lower and upper platform respectively. If the pins shall not precisely fit within their housings, this will mean that the upper platform rotation was not exactly of 60°. In this case the upper platform must be elevated again and the rotation completed until the pins perfectly fit into their housings as it can be seen in Figure 11.

Moreover, besides ensuring a 60° rotation, the pins are located in such a way that they were useful to avoid collisions between the horizontal capacitive sensors and their targets and to maintain the upper platform from shifting as it is being lifted. Furthermore, it must be noted that a tolerance of 1 mm is left between the pins and their housings with the purpose of avoiding the pins from guiding the mechanical positioning of the upper platform with respect to the lower platform, since this must be done by the kinematic couplings.

**Figure 12.**
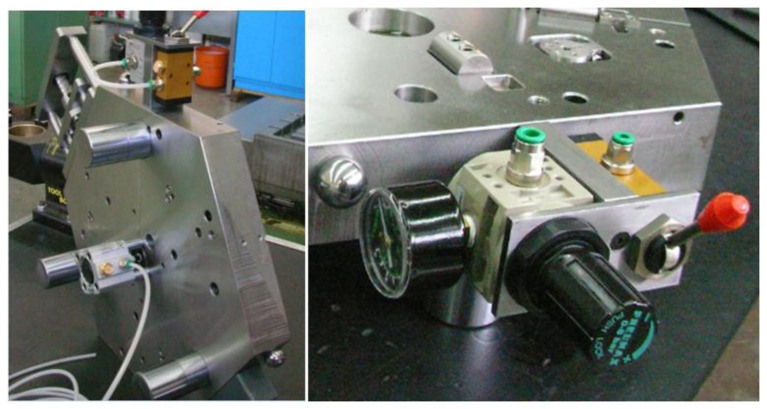
Pneumatic system to generate the kinematic coupling preload.

In the design of kinematic coupling, preload is one of the most important parameters that affect the mechanical repeatability. Preload is the force applied to the coupling to hold it together and establishes the initial stiffness and to get a good stiffness the preload must be high and repeatable. In [26–28,31] this was accomplished by preloading through the center of the kinematic elements with bolts. Furthermore, in our case preload helps to overcome friction and avoid deformations in the contact points between the spheres and cylinders due to the weight of the measuring instrument. Moreover, when mounting a PCMM on the upper platform, the preload force helps to avoid the upper platform from tipping due to the PCMM mass, *i.e.*, an articulated arm coordinate measuring machine. To generate the preload, a pneumatic system fixed to the center shaft of the upper platform was used as shown in Figure 12. The applied pressure to the IMP pneumatic system to generate the preload force was 4 bars.

**Figure 13.**
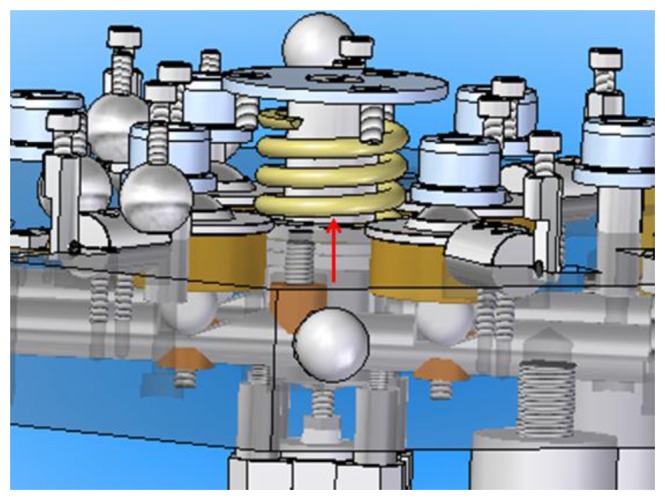
Preload spring used in the Indexed Metrology Platform.

Finally, in order to avoid violent collisions between the spheres and cylinders when the upper platform is descending, a preload spring is attached to the shaft of the upper platform (Figure 13). This spring will be particularly useful when using the IMP with PCMMs of considerable weight such as laser trackers among others.

### 2.3. Utilization of the Capacitive Sensors in the Indexed Metrology Platform

**Figure 14.**
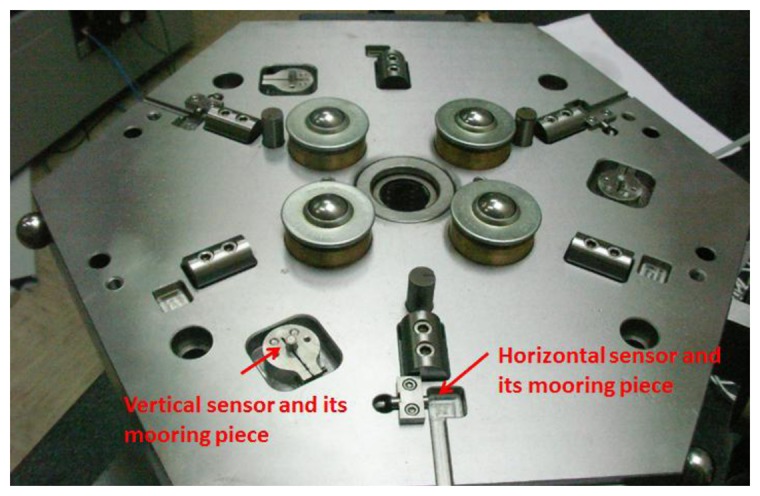
Capacitive sensors with their mooring pieces.

A very important feature in the usage of the IMP during the verification procedure of PCMMs is the capacity of measuring with high precision the position and orientation of the upper platform with respect to the lower platform. To this end, six capacitive sensors with nanometer resolution were used. Three of the sensors were axially located and the other three tangentially located with respect to the rotation axis (shaft) of the IMP. To fix the sensors to the lower platform two different pieces were manufactured for the horizontal and vertical capacitive sensors respectively. During the verification of PCMMs the readings of the capacitive sensors will allow to express coordinates of measured data in the lower platform global coordinate system (GCS) by obtaining the homogenous transformation matrices that link the coordinate system of the upper platform to the GCS of the lower platform for each of the six different positions. In Figure 14 the disposition of the horizontal and vertical sensors as well as the pieces used to fix them to the lower platform are shown.

Capacitive sensors are non-contact measuring devices that use the capacitance electrical property to carry out measurements. A change in distance between the capacitive sensor and its target (an electrical conductor material) produces capacitance changes which in turn generate changes in the sensor's current flow. The sensor's electronic generates a calibrated output voltage proportional to the magnitude of the current flow that allows to know the position of the target. The constant of proportionality of this linear relation between the changes of output voltage and distance is called the sensitivity of the sensor [32–35].

The use of non-contact sensors for measuring small displacements in the range of sub-micrometer and nanometer resolution in applications such as nanopositioning, scanning and metrology has increased considerably in later years. Within this group of sensors, capacitive sensors are becoming more and more used due to their high accuracy and to the insensitivity to changes in a magnetic field [36]. In the field of dimensional metrology applications, three capacitive sensors were used in the Triskelion ultra-precision probe to determine the *X*, *Y* and *Z* deflections of the probe tip of the ISARA 400 [37]. Nevertheless, as far as we know there aren't any references on the use of capacitive sensors in applications with portable coordinate measuring instruments, in particular, coordinate measuring arms or laser trackers.

**Figure 15.**
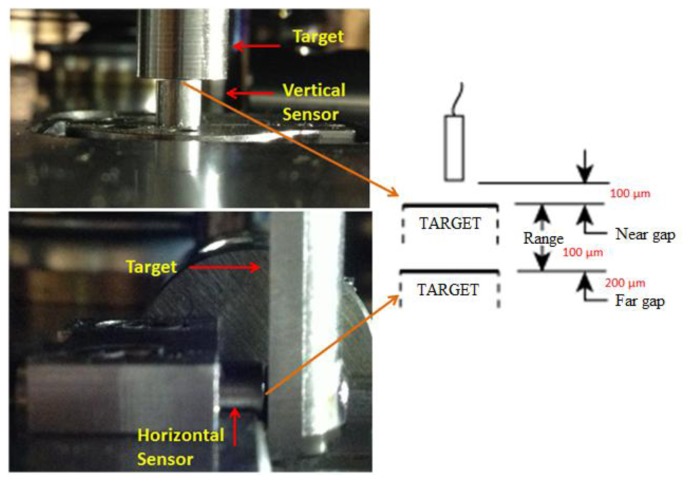
View of the capacitive sensors and targets with their operational working range.

The capacitive sensor model used in the IMP is a C5-E Compact Driver from Lion Precision (Eindhoven, The Netherlands), with a measuring range of 100 μm for an output voltage from 10 to −10 V and an operational range from 100 to 200 μm, where a positive voltage value indicates that the target is between 100 and 150 μm and a negative value indicates that the target is between 150 and 200 μm as it can be seen in Figure 15.

The capacitive sensor's sensitivity provided by the manufacturer was 0.2000 V/μm, which means that for a change of voltage of 0.2 V, a change of 1 μm between the sensor and its target will be observed.

## 3. Evaluation of the IMP Mechanical Repeatability

**Figure 16.**
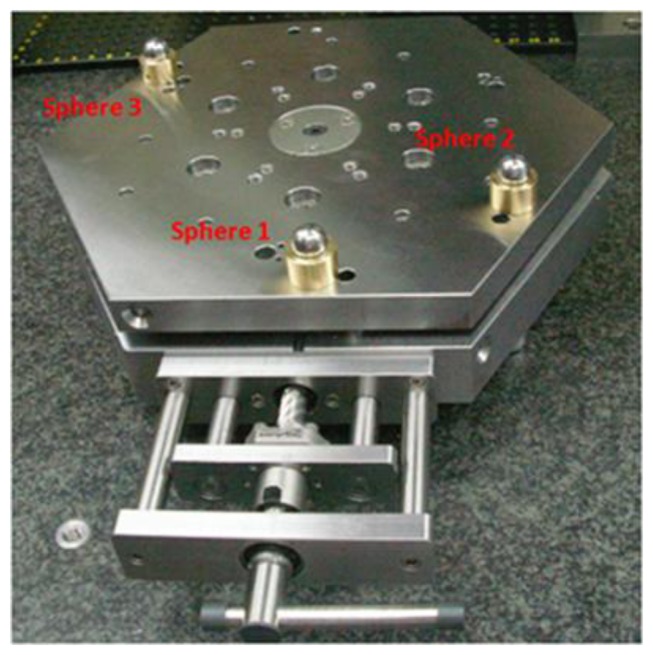
Spheres on upper platform to evaluate the mechanical repeatability of the IMP.

To evaluate the mechanical repeatability of the IMP obtained with the kinematic coupling arrangement three spheres were fixed on top of the upper platform and measured with a CMM (model: PMC 876-CNC, Zeiss, Oberkochen, Germany) as shown in Figure 16.

**Figure 17.**
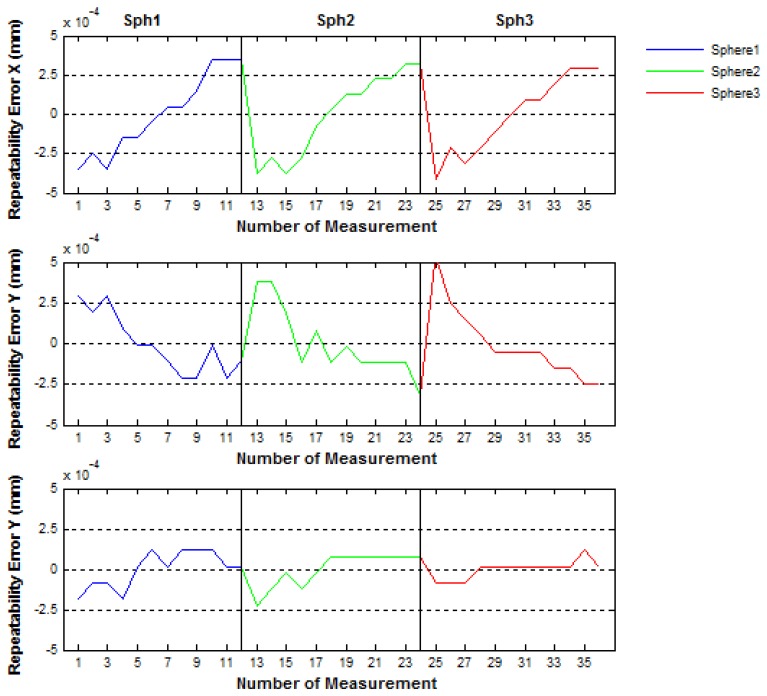
Repeatability errors of the sphere centers measured with the CMM.

Firstly, it was important to verify the achievable precision with the CMM. To this end, the centers of the spheres 1, 2 and 3 were measured twelve times without elevating the upper platform. The spheres were measured in ascending order, starting with sphere 1 and ending with sphere 3 in each of the six positions of the IMP. The repeatability error was obtained from calculating the coordinates of the sphere center as the mean of the twelve data in each of the *X*, *Y* and *Z* coordinates. For the *X* coordinate the following equation is used (Equation (9)):

(9) $${\bar{X}}_{i}=\frac{\sum_{m=1}^{n_{i}}X{\left(m\right)}_{i}}{n_{i}}$$

where *n_i_* is the number of data captured, 12 in this case, for sphere *i, i* = *1,2,3.* Analogously for the *Y* and *Z* coordinates. The repeatability errors observed were less than 0.7 μm as it can be observed in Figure 17.

**Figure 18.**
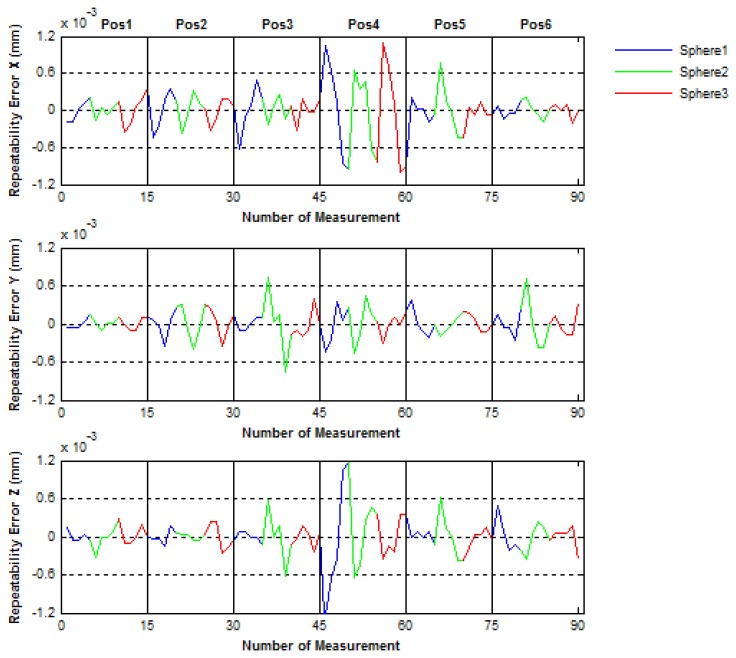
Positioning mechanical repeatability errors of the platform for the No-Continuous way.

**Figure 19.**
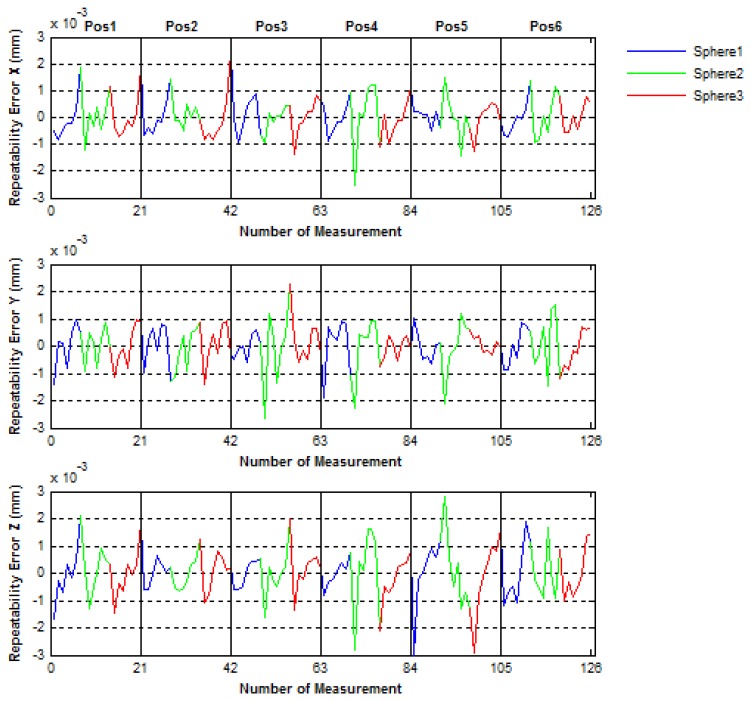
Positioning mechanical repeatability errors of the platform for the Continuous way.

Then, to evaluate the positioning mechanical error of the IMP, the spheres centers were measured five times in two different ways. In the first way (called No-Continuous) the center of the three spheres were measured five times in each IMP position before turning 60° to the next position, in such a way that the all measuring procedure is done with a complete turn of 360° of the platform. Inversely, in the second way (called Continuous), after a turn of 60° in each position one measurement of the spheres centers is performed, in such way that to complete the all measuring procedure implies to realize five complete turns of 360° of the platform. In Figures 18 and 19 the mechanical positioning errors for both No-Continuous and Continuous are presented. The figures show that the errors obtained were 2.4 μm and 4 μm for both the No-Continuous and Continuous ways, respectively and that the positioning errors are acceptable in order to use the IMP in the verification procedures of PCMMs.

The importance of the positioning mechanical repeatability resides in the necessity of maintaining the six capacitive sensors within their working range (100–200 μm) in all six different platform positions in order to use it in the verification procedures of PCMMs. With the observed maximum mechanical repeatability error, all six sensors stay within their working range in the six platform positions.

## 4. Characterization of the Capacitive Sensors Used in the IMP

**Figure 20.**
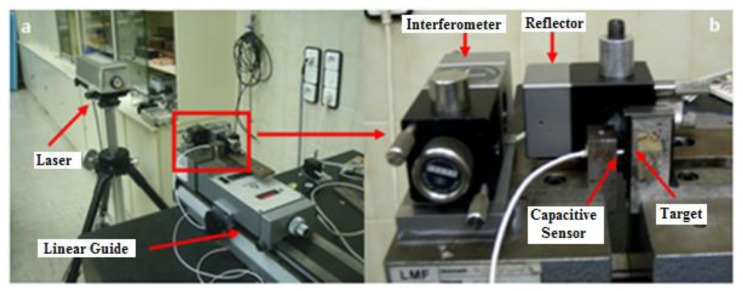
Experimental setup of sensor's characterization tests.

As mentioned before, the capacitive sensors will allows us to precisely know the position and orientation of the upper platform with respect to the lower platform, so it is of great importance to verify that the sensors meet the specifications provided by the manufacturer. To this end, a characterization of the capacitive sensors was performed. The characterization was carried out using a laser interferometer (HP 5528A, Hewlett-Packard, Palo Alto, CA, USA) as a calibration device. The interferometer, reflector, capacitive sensor and capacitive sensor's target were fixed to a linear guide, with the reflector and capacitive sensor's target located on the moving part of the guide as shown in Figure 20. This way, both the reflector and target moved simultaneously, making it possible to record the laser interferometer and capacitive sensor readings and then compare them.

In an effort to cover most of the capacitive sensor's calibrated area (100–200 μm), tests were carried out inside the calibrated area of the sensor, taking 10,000 data every 10 μm to calculate the sensitivity, sensitivity error and linearity error. To calculate the capacitive sensor's repeatability value, 300,000 data points were taken at three different locations inside the calibrated area of the sensor. The results obtained are compared when possible to the calibration sheet report obtained from Lion Precision. The sensitivity and sensitivity error calculated for the capacitive sensor were 0.2012 V/μm (0.2 V/μm Lion Precision data sheet) and 0.1497% respectively. The overall linearity of the capacitive sensor showed to be linear to within 0.09% (0.03% Lion Precision data sheet) over the entire operational measurement range covered by the sensor. Regarding the repeatability of the sensor, the repeatability values in each of the three locations (6V, 0V, −6V) were 0.010572, 0.008333 and 0.006147 μm, respectively.

By analyzing the sensor characterization results, it was possible to conclude that the capacitive sensors met the specifications provided by the manufacturer and thus their suitability for using them in the IMP.

## 5. Capacitive Sensor Mathematical Model in an AACMM Verification Procedure Using the IMP

To use the IMP in the verification procedures of PCMMs, a mathematical model linking the measurement instrument (*i.e.*, AACMM) coordinate system with the fixed global coordinate system of the platform needs to be determined. This model uses the capacitive sensors readings from the verification procedure and the optimal geometrical parameters found during the IMP calibration procedure. It must be noted that throughout the verification procedure it is not possible to measure the characterization spheres of the upper and lower platforms with a CMM, so the mathematical model must allow us to find a homogenous transformation matrix (HTM) that expresses the AACMM *x*, *y* and *z* readings in the fixed global coordinate system of the lower platform for each of the six IMP different positions [38]. To this end, besides the optimal geometrical parameters, reference saved data from the IMP calibration procedure is also used in this mathematical model [38]. The reference data used in the verification is the following:

- Reference HTM from calibration procedure (reference matrix);
- Geometrical intersection point between each sensor ant its target in each of the six different IMP positions (reference point);
- Capacitive sensors readings that correspond to the reference HTM.

**Figure 21.**
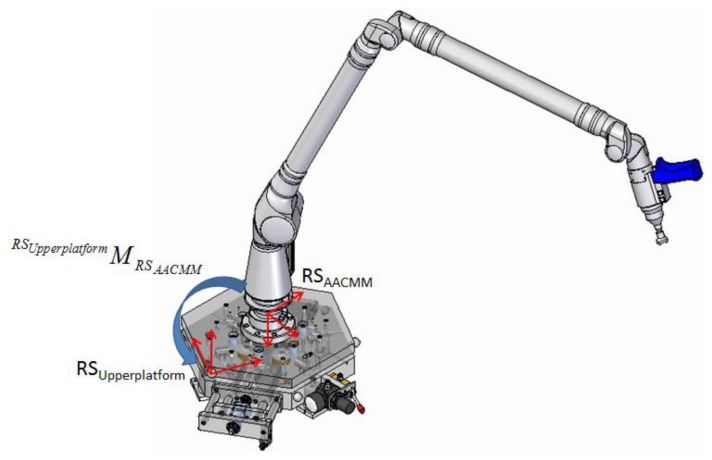
Reference systems of the AACMM and upper platform.

**Figure 22.**
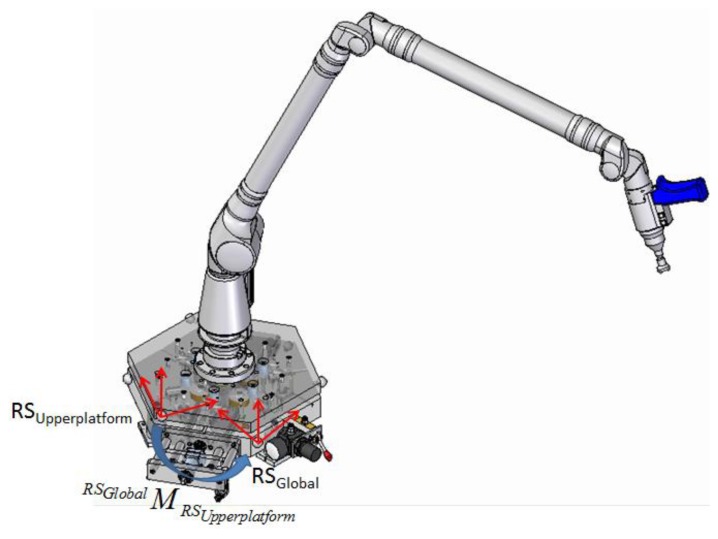
Reference system of the upper platform and fixed lower platform.

This way, the steps to carry out the verification procedure will basically consist of:

1. Mount the AACMM on the IMP as shown in Figure 21.
2. Positioning of IMP in its position 1. The IMP six positions are marked so that the operator exactly knows the IMP current position. Measure the upper platform characterization spheres with the measuring arm to determine the HTM (^RSupperplatform^M_RS_AACMM_) that links the AACMM reference system with the upper platform reference system as shown in Figure 21. It is extremely important that the upper platform characterization spheres are measured in the same order that they were measured during the calibration procedure of the IMP. Moreover, since the measuring arm is fixed to the upper platform, their reference systems have solidary movements between them, so it is only necessary to determine the HTM between systems only once.
3. Find the current reference system geometrical relationship of position 1 with respect to the fixed global reference system (lower platform) by means of the capacitive sensors readings during the verification procedure and the optimal geometrical parameters obtained from the IMP calibration procedure.
4. Measurement of the *n* spheres of the ball bar gauge fixed in a position and orientation around the AACMM measuring volume. The spheres measured in our case are presented in Figure 25b, below.
5. Obtain the geometric relationship between the upper platform and fixed lower platform reference systems for all the measured points on the *n* spheres of the ball bar gauge. Since the upper platform rests on the fixed lower platform by means of the kinematic couplings, when the measuring arm moves to reach and probe a sphere, a dynamic force is generated. This movement could cause micro deformations in the configuration of kinematic couplings used. These micro deformations can change the position of the upper platform with respect to the fixed lower platform, in which case, this position will not be the same every time that a point on a sphere is probed with the measuring arm, making it necessary to determine a different HTM for every probed point as shown in Figure 22.
6. Turn the upper platform to position 2 and determine the geometric relationship between the current reference system with the global reference system of the fixed platform using the capacitive sensors readings and the set of optimum geometrical parameters data from the calibration procedure.
7. Measure the *n* spheres of the ball bar gauge.
8. As in step 5, obtain the geometrical relationship between the upper platform and fixed lower platform reference systems for all the measured points on the *n* spheres of the ball bar gauge in position 2.
9. Repeat steps 6, 7 and 8 for each of the four remaining positions of the platform.

**Figure 23.**
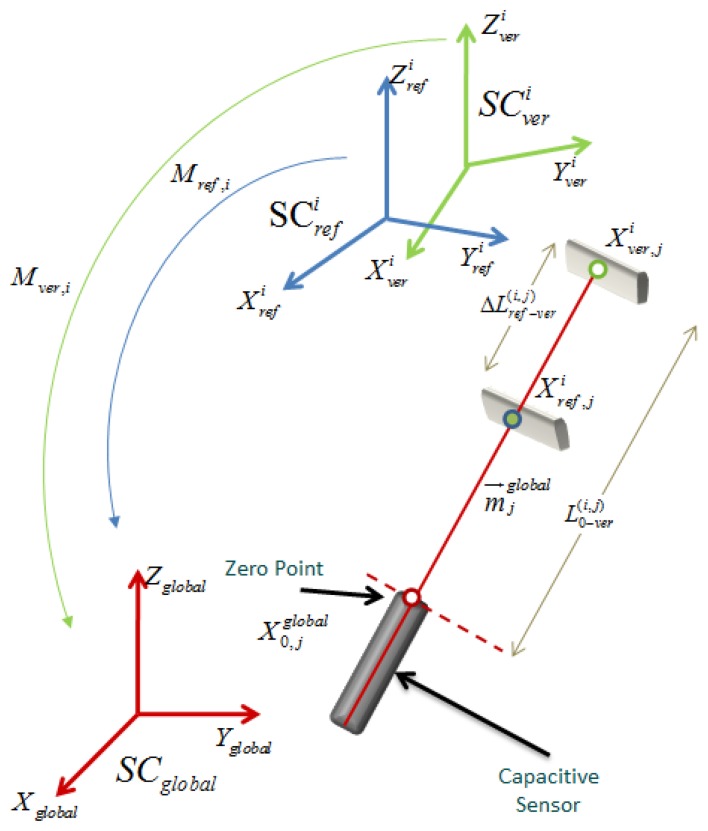
Capacitive sensor geometric scheme between the reference position from the calibration procedure and a new platform positioning during the verification procedure.

The mathematical model that allows us to find the HTMs to express the probed points in the fixed global coordinate system, using the capacitive sensors readings from the verification procedure and the set of parameters from the calibration procedure is explain. In Figure 23 a geometric scheme of a capacitive sensor between the reference position obtained from the calibration procedure and the platform position during the verification procedure is shown.

The notation that corresponds to Figure 23 that is used to develop the mathematical model is described next:

- *SC_global_*: Fixed global reference system [*X_global_*, *Y_global_*, *Z_global_*] of the lower platform.
- $SC_{\text{ref}}^{i}$: Reference system [ $X_{\text{ref}}^{i}$, $Y_{\text{ref}}^{i}$, $Z_{\text{ref}}^{i}$], of the upper platform position *i*, for *i* = *1,2,*…*,6*, determined during the calibration procedure.
- $SC_{\text{ver}}^{i}$: Reference system determined in the verification procedure [ $X_{\text{ver}}^{i}$, $Y_{\text{ver}}^{i}$, $Z_{\text{ver}}^{i}$], of the upper platform position *i*, for *i* = *1,2,*…*,6*.
- *M_ref,i_*: Reference HTM that allows to express readings from the $SC_{\text{ref}}^{i}$ of position *i* to *SC_global_*, for *i* = *1,2,*…*,6*, which is obtained during the calibration procedure.
- *M_ver,i_*: Search HTM of position *i*, that allows to express readings from $SC_{\text{ver}}^{i}$ to SC_global_, for *i* = *1,2,*…,*6*.
- $X_{0,j}^{\text{global}}$: Capacitive sensor zero point vector [ $x_{0,j}^{\text{global}}$, $y_{0,j}^{\text{global}}$, $z_{0,j}^{\text{global}}$, 1]*^T^* expressed in the global reference system, *SC_global_*, for *j* = *1,2,*…*,6* capacitive sensors.
- $X_{\text{ref},j}^{i}$: Vector [ $x_{\text{ref},j}^{i}$, $y_{\text{ref},j}^{i}$, $z_{\text{ref},j}^{i}$, 1]*^T^* of the target point from the calibration reference position corresponding to sensor *j* in position *i*, for *j* = *1,2,*…*,6* and *i* = *1,2,*…*,6*, expressed in the $SC_{\text{ref}}^{i}$ reference system, obtained from the calibration procedure.
- $X_{\text{ver},j}^{i}$: Vector [ $x_{\text{ver},j}^{i}$, $y_{\text{ver},j}^{i}$, $z_{\text{ver},j}^{i}$, 1]*^T^* of the target point from the verification procedure new position corresponding to sensor *j* in position *i*, for *j* = *1,2,*…*,6* and *i* = *1,2,*…*,6*, expressed in the $SC_{\text{ver}}^{i}$ reference system.
- $m_{j}^{\text{global}}$: Measuring direction cosine vector of the capacitive sensors, [ $\cos\alpha_{j}^{\text{global}}$, $\cos\beta_{j}^{\text{global}}$, $\cos\gamma_{j}^{\text{global}}$, 0]*^T^* for *j* =*1,2,*…*,6* capacitive sensors, expressed in the *SC_global_* reference system.
- $\Delta L_{\text{ref}-\text{ver}}^{\left(i,j\right)}$: Difference between $L_{\text{ref}}^{\left(i,j\right)}$, value of the capacitive sensor *j* (in the reference position *i*), and $L_{\text{ver}}^{\left(i,j\right)}$, value of the capacitive sensor *j* in position *i* (of a new position) in the verification procedure.
- $L_{0-\text{ver}}^{\left(i,j\right)}$: Value of the capacitive sensor *j* (in the reference position *i*) in the verification procedure.

As mentioned before, the final objective of the verification procedure mathematical model is to determine the HTM that links the upper platform reference system with the fixed global reference system in each of the platform six positions for all the measuring arm probed points. This way, taking position 1 of the platform as an example, we can formulate a mathematical model based on the optimized geometric features (calculated in the calibration procedure of IMP), the set of saved data from the reference position of the ith-position (reference coordinate system ( $SC_{\text{ref}}^{i}$), the reference HTM, vector of the reference point, capacitive sensors readings) and the capacitive sensors readings obtained during the verification procedure for position 1. This model of nonlinear system of equations has as unknowns the searched HTM parameters (*M_ver,1_*):

(10) $$M_{\text{ver},1}X_{\text{ver},j}^{1}=M_{\text{ref},1}X_{\text{ref},j}^{1}+\Delta L_{\text{ref}-\text{ver}}^{\left(1,j\right)}{\vec{m}}_{j}^{\text{global}}$$

(11) $$M_{\text{ver},1}X_{\text{ver},j}^{1}=X_{\text{ver},j}^{\text{global}}=X_{0,j}^{\text{global}}+L_{0-\text{ver}}^{\left(1,j\right)}{\vec{m}}_{j}^{\text{global}}$$

By denoting:

(12) $$T_{\text{ref}-\text{ver}}^{\left(1,j\right)}=\left[\begin{array}{rrrr} 1 & 0 & 0 & \Delta L_{\text{ref}-\text{ver}}^{\left(1,j\right)}\cos\alpha_{j}^{\text{global}}\\ 0 & 1 & 0 & \Delta L_{\text{ref}-\text{ver}}^{\left(1,j\right)}\cos\beta_{j}^{\text{global}}\\ 0 & 0 & 1 & \Delta L_{\text{ref}-\text{ver}}^{\left(1,j\right)}\cos\gamma_{j}^{\text{global}}\\ 0 & 0 & 0 & 0 \end{array}\right]$$

and:

(13) $$T_{0-\text{ver}}^{\left(1,j\right)}=\left[\begin{array}{rrrr} 1 & 0 & 0 & \Delta L_{0-\text{ver}}^{\left(1,j\right)}\cos\alpha_{j}^{\text{global}}\\ 0 & 1 & 0 & \Delta L_{0-\text{ver}}^{\left(1,j\right)}\cos\beta_{j}^{\text{global}}\\ 0 & 0 & 1 & \Delta L_{0-\text{ver}}^{\left(1,j\right)}\cos\gamma_{j}^{\text{global}}\\ 0 & 0 & 0 & 0 \end{array}\right]$$

and substituting $\Delta L_{\text{ref}-\text{ver}}^{\left(1,j\right)}{\vec{m}}_{j}^{\text{global}}$ in Equation (12) by $T_{\text{ref}-\text{ver}}^{\left(1,j\right)}$ and $L_{0-\text{ver}}^{\left(1,j\right)}{\vec{m}}_{j}^{\text{global}}$ in Equation (13) by $T_{0-\text{ver}}^{\left(1,j\right)}$ we obtain the following equivalent equations:

(14) $$M_{\text{ver},1}X_{\text{ver},j}^{1}=T_{\text{ref}-\text{ver}}^{\left(1-f\right)}M_{\text{ref},1}X_{\text{ref},1}^{1}=Y_{\text{ref}-\text{ver}}^{\left(1,j\right)}$$

(15) $$M_{\text{ver},1}X_{\text{ver},j}^{1}=T_{0-\text{ver}}^{\left(1-j\right)}X_{0,j}^{\text{global}}=Y_{0-\text{ver}}^{\left(1,j\right)}$$

This way, by taking $x={\left(M_{\text{ver},1}^{11},M_{\text{ver},1}^{12},\ldots ,M_{\text{ver},1}^{44},x_{\text{ver},1}^{1},y_{\text{ver},1}^{1},z_{\text{ver},1}^{1},\ldots ,x_{\text{ver},6}^{1},y_{\text{ver},6}^{1},z_{\text{ver},6}^{1}\right)}^{T}$ as the vector of unknowns that contain the search HTM, the following non-linear system of equations can be expressed:

(16) $$F(x)=\left(\begin{array}{c} M_{\text{ver},1}X_{\text{ver},1}^{1}-Y_{\text{ref}-\text{ver}}^{\left(1,1\right)}\\ \vdots \\ M_{\text{ver},1}X_{\text{ver},6}^{1}-Y_{\text{ref}-\text{ver}}^{\left(1,6\right)}\\ M_{\text{ver},1}X_{\text{ver},1}^{1}-Y_{0-\text{ver}}^{\left(1,1\right)}\\ \vdots \\ M_{\text{ver},1}X_{\text{ver},6}^{1}-Y_{0-\text{ver}}^{\left(1,6\right)} \end{array}\right)=0$$

that can be solved by means of Levenberg-Marquardt algorithm using the Matlab software.

The HTM found must be an orthonormal matrix that meets the conditions of orthogonality and normality. To meet these conditions the following constraints are included in the mathematical algorithm:

(17) $$M_{\text{ver},1}^{11}*M_{\text{ver},1}^{12}+M_{\text{ver},1}^{21}*M_{\text{ver},1}^{22}+M_{\text{ver},1}^{31}*M_{\text{ver},1}^{32}=0$$

(18) $$M_{\text{ver},1}^{11}*M_{\text{ver},1}^{13}+M_{\text{ver},1}^{21}*M_{\text{ver},1}^{23}+M_{\text{ver},1}^{31}*M_{\text{ver},1}^{33}=0$$

(19) $$M_{\text{ver},1}^{12}*M_{\text{ver},1}^{13}+M_{\text{ver},1}^{22}*M_{\text{ver},1}^{23}+M_{\text{ver},1}^{32}*M_{\text{ver},1}^{33}=0$$

(20) $${\left(M_{\text{ver},1}^{11}\right)}^{2}+{\left(M_{\text{ver},1}^{21}\right)}^{2}+{\left(M_{\text{ver},1}^{31}\right)}^{2}=1$$

(21) $${\left(M_{\text{ver},1}^{12}\right)}^{2}+{\left(M_{\text{ver},1}^{22}\right)}^{2}+{\left(M_{\text{ver},1}^{32}\right)}^{2}=1$$

(22) $${\left(M_{\text{ver},1}^{13}\right)}^{2}+{\left(M_{\text{ver},1}^{23}\right)}^{2}+{\left(M_{\text{ver},1}^{33}\right)}^{2}=1$$

Equations (17)–(19) and (20)–(22) meet the conditions of orthogonality and normality of the HTM found respectively.

**Figure 24.**
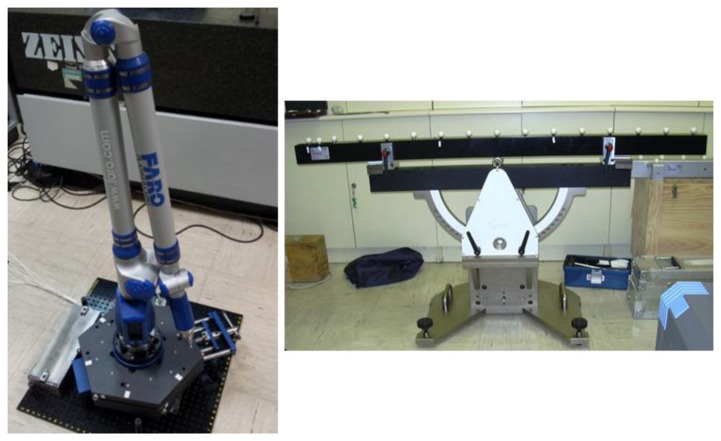
Faro Platinum measuring arm mounted on IMP and ball bar calibrated gauge used in the verification.

To verify the correct functioning of the platform, a volumetric verification of an AACMM was carried out based on the ASME B89.4.22 norm. The AACMM used in the volumetric verification is a Faro Platinum (Figure 24) with a diameter measuring volume of 2.4 meters and a 2-2-3 measuring configuration type and a volumetric precision reported by the manufacturer of ± 0.043 mm. The calibrated gauge object used consists of a ball bar gauge with the distances between the spheres centers calibrated.

**Figure 25.**
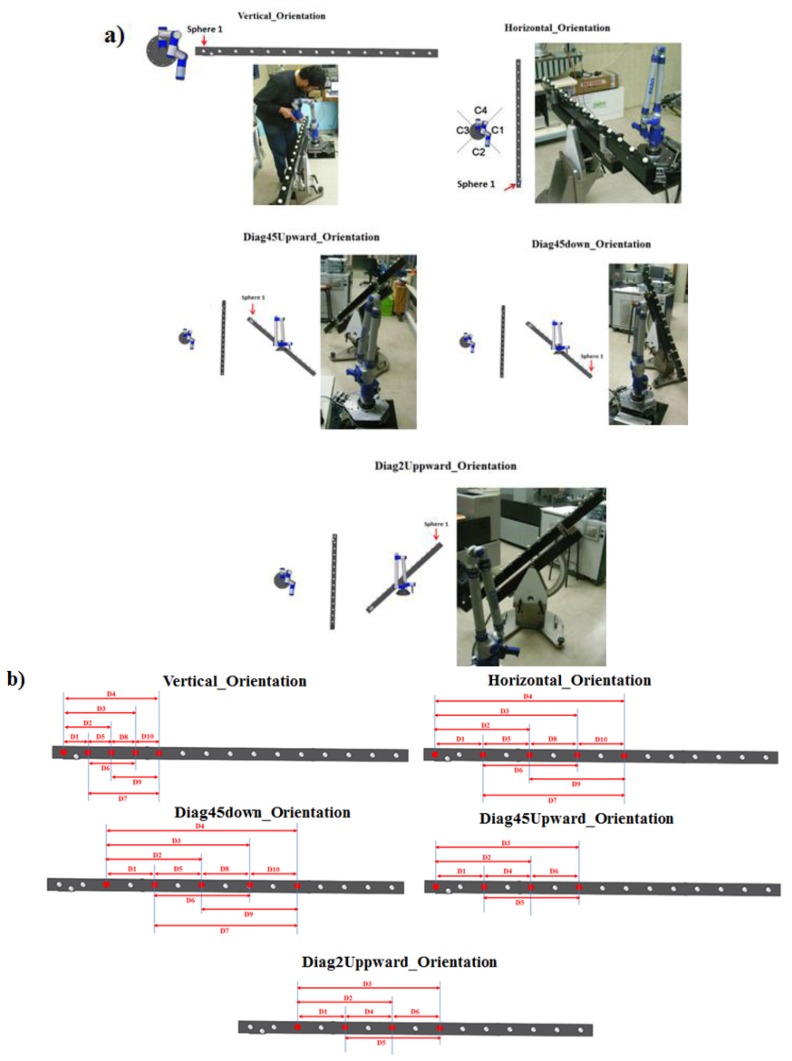
(**a**) Orientations of the ball bar gauge selected for the verification procedure; (**b**) Materialized distances between spheres centers measured.

The ASME B89.4.22 volumetric test recommends locating the ball bar gauge in 20 different orientations around the AACMM in order to evaluate most of its measuring volume. In this work we selected five orientations from the 20 recommended by the norm. Nevertheless, it must be noted that by evaluating these five orientations in the six positions of the platform will be the equivalent to locating the ball bar gauge in 30 different orientations with respect to the AACMM, more than the ones suggested by the norm which allows to evaluate a greater measuring volume in a significant less time and physical effort. The orientations of the ball gauge bar and the spheres measured in each orientation are presented in Figure 25.

**Figure 26.**
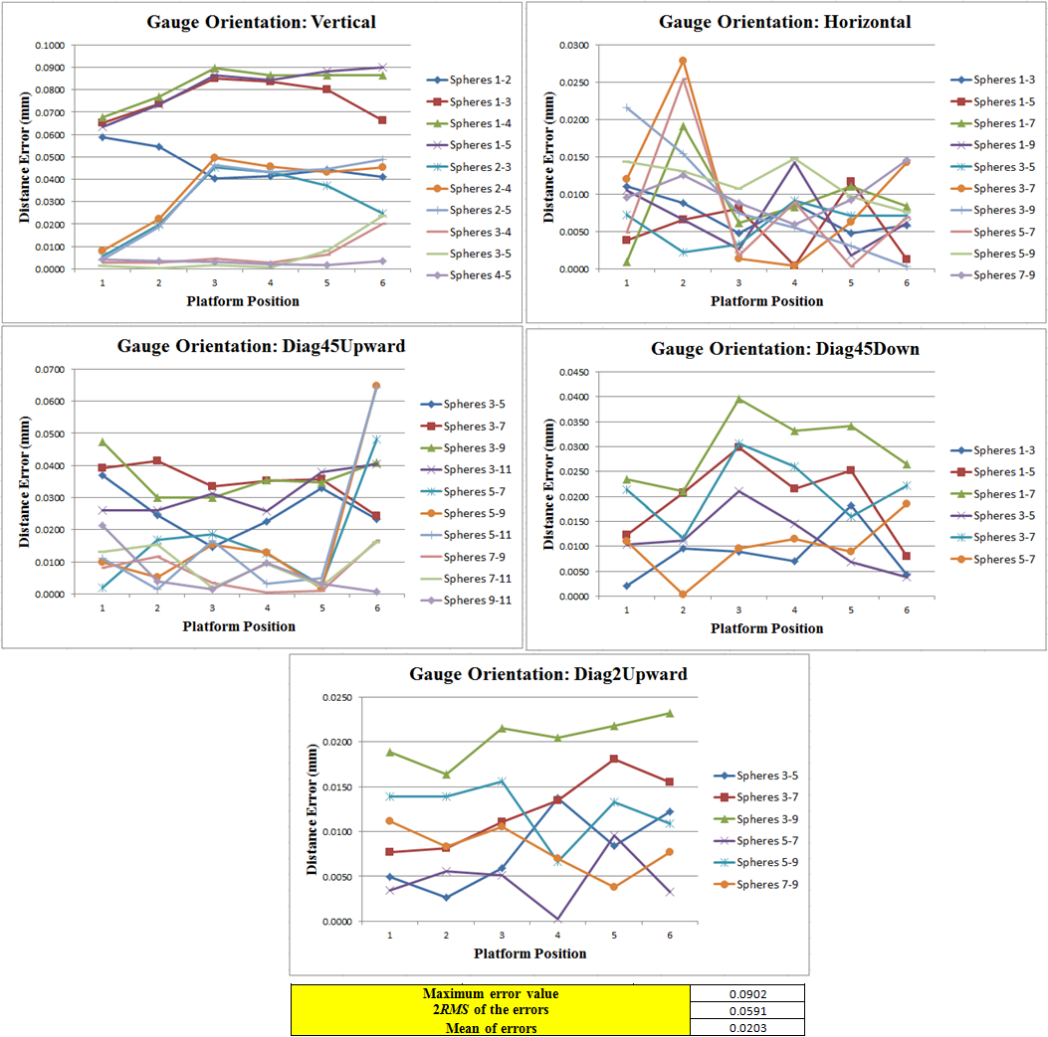
Maximum distance error graphs and two times the root mean square of the 252 distances between sphere centers (mm).

Since in three of the orientations of the ball bar gauge five spheres were measured and in the remaining ones four spheres were measured, we were able to materialize 252 distances between centers (for all six platform positions) and compared them to the nominal distances to obtain 252 distance errors. These errors were used to evaluate the measuring volume of the AACMM by calculating the root mean square value. The results are shown in Figure 26.

In Figure 26 it can be observed that the maximum distance error out of the 252 errors is 90.2 μm that corresponds to the position 6 of the platform in the vertical ball bar gauge orientation between spheres 1 and 5; the mean of the errors is 20.3 μm and the value of two times the root square is 59.1 μm. According to these results we can conclude that the volumetric precision for the AACMM evaluated is the proper one for a measuring instrument of its characteristics. Moreover, by using the platform we simplified the time required to carry out these type of procedure from over a working day to three hours as well as greatly reducing the physical effort. Finally it is important to mention that the results are comparable to the results obtained without using the platform, which ensures the correct functioning of the platform and the mathematical algorithms used.

## 6. Conclusions

In this work the mechanical design and the most important mechanical components of the elevation, rotation and descent mechanism of the IMP are explained in detail. The arrangement of the kinematic coupling used to cancel the degrees of freedom of the upper platform with respect to the lower platform and to obtain a high mechanical positioning repeatability is presented. Moreover, the determination of the parameter values of the kinematic couplings are determined based on the angle in which the contact force vectors intersect the plane of coupling action. In regards to the elevation mechanism, the slope inclination of the elevation bars was determined as a function of the upper platform weight and the approximately weight of a measuring instrument. Additionally, the importance of the preload force to get a good stiffness in the coupling arrangement and the pneumatic system fixed to the upper platform used to generate the preload force are explained.

Furthermore, the assessment of the positioning mechanical error repeatability of the indexed metrology platform is obtained in two different ways. In both ways the maximum positioning mechanical error was less than 4 μm, which allow us to conclude that the positioning errors are acceptable in order to use the IMP in the verification procedures of PCMMs.

A very important issue in order to use the IMP in the verification procedure is the use of six capacitive sensors to exactly determine the position and orientation of the upper platform with respect to the lower platform. To this end, a characterization of the capacitive sensors was carried out and the results compared to the specifications provided by the manufacturer. The sensitivity of the sensor obtained was 0.2012 V/μm in comparison to the 0.2 V/μm provided in the Lion Precision data sheet, so we were able to conclude that the capacitive sensors met the specifications provided by the manufacturer and thus their suitability for using them in the IMP.

Finally the mathematical model based on the capacitive sensors readings during the verification procedures is explained; and the results of an AACMM volumetric assessment using the indexed metrology platform are discussed, highlighting the drastically simplification in both time and effort when carrying out these type of procedures.

## Figures and Tables

**Table 1.** Different angle *ϕ* values evaluated and their corresponding forces.

**Angle (*ϕ*)**	**Linear Displacement of Steel Bars (*mm*)**	**Force Needed to Elevate One Sphere (*N*)**
20	17.56	48.095
30	13.04	69.794
40	10.39	110.229
45	9.6	149.011
50	8.96	224.199
60	8.01	7,379.72
